# Setting priorities in child health research in India for 2016-2025: a CHNRI exercise undertaken by the Indian Council for Medical Research and INCLEN Trust

**DOI:** 10.7189/jogh.09.020701

**Published:** 2019-12

**Authors:** Kerri Wazny, Narendra K Arora, Archisman Mohapatra, Hema S Gopalan, Manoj K Das, MKC Nair, Sandeep Bavdekar, Reeta Rasaily, Vasantha Thavaraj, Malabika Roy, Chander Shekhar, Rakesh Kumar, Vishwa M Katoch, Igor Rudan, Robert E Black, Soumya Swaminathan

**Affiliations:** 1 Centre for Global Health Research, Usher Institute of Population Health Sciences and Informatics, University of Edinburgh, Scotland, UK; 2 The INCLEN Trust International, New Delhi, India; 3 Kerala University of Health Sciences, Thrissur, Kerala, India; 4 Department of Pediatrics, ‎Topiwala National Medical College and BYL Nair Charitable Hospital, Mumbai, Maharashtra, India; 5 The Indian Council of Medical Research, New Delhi, India; 6 Johns Hopkins Bloomberg School of Public Health, Baltimore, Maryland, USA; 7 World Health Organization, Geneva, Switzerland**; * Joint first authors; ** Formerly The Indian Council of Medical Research, New Delhi, India)

## Abstract

**Background:**

Millennium Development Goal 4 (MDGs) mobilised countries to reduce child mortality by two thirds the 1990 rate in 2015. While India did not reach MDG 4, it considerably reduced child mortality in the MDG-era. Efficient and targeted interventions and adequate monitoring are necessary to further progress in improvements to child health. Looking forward to the Sustainable Development Goal (SDG)-era, the Indian Council of Medical Research and The INCLEN Trust International conducted a national research priority setting exercise for maternal, child, newborn health, and maternal and child nutrition. Here, results are reported for child health.

**Methods:**

The Child Health and Nutrition Research Initiative (CHNRI) method for research priority setting was employed. Research ideas were crowd-sourced from a network of child health experts from across India; these were refined and consolidated into research options (ROs) which were scored against five weighted criteria to arrive weighted Research Priority Scores (wRPS). National and regional priority lists were prepared.

**Results:**

90 experts contributed 596 ideas that were consolidated into 101 research options (ROs). These were scored by 233 experts nationwide. National wRPS for ROs ranged between 0.92 and 0.51. The majority of the top research priorities related to development of cost-effective interventions and their implementation, and impact evaluations, improving data quality; and monitoring of existing programs, or improving the management of morbidities. The research priorities varied between regions, the Economic Action Group and North-Eastern states prioritised questions relating to delivering interventions at community- or household-level, whereas the North-Eastern states and Union Territories prioritised research questions involving managing and measuring malaria, and the Southern and Western states prioritised research questions involving pharmacovigilance of vaccines, impact of newly introduced vaccines, and delivery of vaccines to hard-to-reach populations.

**Conclusions:**

Research priorities varied geographically, according the stage of development of the area and mostly pertained to implementation sciences, which was expected given diversity in epidemiological profiles. Priority setting should help guide investment decisions by national and international agencies, therefore encouraging researchers to focus on priority areas. The ICMR has launched a grants programme for implementation research on maternal and child health to pursue research priorities identified by this exercise.

Globally, 5.4 million children die every year, many due to preventable causes [1]. One fifth of these deaths occur in just one country: India [2]. Effective and locally appropriate interventions with high coverage and quality have the potential to substantially reduce these deaths.

The Millennium Development Goal era (MDG) resulted in large reductions in child mortality in numerous countries worldwide; the MDGs were a powerful advocacy tool as they provided an agenda with succinct, measurable goals [3]. MDG 4 aimed to reduce child mortality by two-thirds from 1990 to 2015. While India did not meet this goal, it achieved considerable reductions in child mortality, especially in infectious causes such as diarrhoea, lower respiratory tract infections, and tuberculosis [4]. Child deaths in India have reduced from 126 deaths per thousand live births in 1990 to 39 deaths per thousand live births in 2017, with an annual rate of reduction of 4.3% [1].

The mortality reductions have not been uniform across the country [2,5]. A recent study demonstrated that if all of India had matched the progress of some high-achieving states, India would have met the MDG targets [2]. The same study showed variations in mortality between poorer and richer states, and in rates of mortality reduction among urban vs rural areas [2]. It is therefore important to prioritise and target interventions at the regional as well as the national level. Moreover, at the completion of the MDGs, the Countdown Report found that equity gaps have persisted in key interventions such as immunisations and that access to improved sanitation is a challenge in rural communities [6]. Prioritizing preventive interventions like these could not only help in reducing mortality but also in ensuring children to attain optimal developmental milestones and achieve their full lifetime potential.

In the beginning of the MDG-era, criticisms included the poor correlation of research funding with the conditions resulting in the greatest mortality and disability, and with the types of research that could have the most immediate impact. The so-called 10/90 gap showed that 90% of research funding went to conditions that accounted for 10% of the global disease burden [7-10]. Advocacy to reduce this gap, and for support of implementation research in low- and middle-income country (LMIC) contexts may have influenced progress. The results of research prioritisation exercises using the Child Health and Nutrition Research Initiative (CHNRI) method overwhelmingly prioritised research for health systems and implementation research that have immediate usability over more flashy, ‘discovery’ research that could take years for translation into programmatic action and impact [8,10]. Thus, research prioritisation has evolved over time as a planning and advocacy tool for directing funding, as well as guiding the work of researchers.

With the knowledge that India was unlikely to meet MDG 4, looking forward to the Sustainable Development Goal (SDG) era, and being aware of the importance of targeting and prioritising research options, especially in a context of resource constraints, the Indian Council of Medical Research (ICMR) and the INCLEN Trust International (INCLEN) began a national research priority setting exercise using the CHNRI method in 2012 [11]. In this paper, which is one of four thematic papers in a series on research priorities for (i) maternal, (i) child and (iii) newborn health and (iv) maternal and child nutrition in India, we describe the results of this exercise for the child health theme.

## METHODS

The CHNRI method is the dominant method of health research priority setting [12]. It capitalises on experts’ wisdom to generate and score research priorities against pre-selected criteria, and is a transparent and systematic method to set research priorities [10]. Below, we have provided a succinct account of our methods; however, the detailed methodology the methods used in the ICMR-INCLEN national research priority setting exercise is published elsewhere [13].

A multidisciplinary and multi-stakeholder national steering group (NSG), comprised of policy makers, academics, program managers, civil society representatives, and donors was assembled to oversee the exercise. The NSG finalized 12 areas of concern (AOC) for the child health theme that together accounted for 75% of child mortality and morbidity. A child health research sub-committee (RSC) was set up and included 26 experts that further developed the technical details for this theme, including the iterative refinement and consolidation of the research options. Further information on their involvement is published elsewhere [13].

A nation-wide network of 186 experts was established [13]; they were located in the various states and union territories to capture diverse programmatic, socio-cultural, and geographic contexts within the country. These experts along with the 26 RSC members were invited to contribute to the first step of the CHNRI exercise which entailed crowd-sourcing research questions (RQs), refined into research ideas (RIs). These RIs were iteratively consolidated into research options (ROs). In the second step of crowd sourcing, ROs were scored using criteria pre-defined.

Five criteria were selected for scoring: (i) relevance; (ii) answerability; (iii) equity; (iv) innovation and investments in research resolving complex and refractory challenges; and, (v) investment in research. Experts were assigned to score two of the five criteria to reduce scorer fatigue [13]. Scorers were asked to score 0 if the research question does not satisfy the criterion, or 1, if the research question satisfied the criterion. Research Priority Score (RPS), which is the mean score across each criterion divided by 100, were calculated, and these scores could range from 0.00 to 1.00. Average Expert Agreement (AEA) were also computed for each research option. The AEA is calculated as follows:


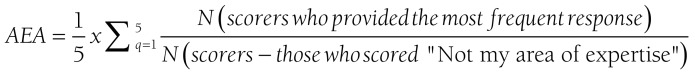


In tandem with the scoring phase of the CHNRI exercise, a Larger Reference Group (LRG), which contained diverse stakeholder groups including politicians, policy makers, funding agencies, and senior researchers was created and tasked with weighting the criteria against one another. This exercise is described fully elsewhere [13]. The resulting weights were applied to each criterion prior to calculating the RPS, resulting in a weighted RPS (wRPS). The ROs were ranked in descending order of their wRPS. Finally, three of the authors (KW, AM, and HG) reviewed the ROs by their domains to classify them. Any disagreements were resolved by a senior researcher (NA).

India is a large country with diverse population and heterogeneous governance status. We, therefore presented national research priorities, as well as regional priorities. The states were grouped into the following regions: (i) EAG (Empowered Action Group) and North-Eastern (NE) states; (ii) Northern states and Union territories (UTs) (including West Bengal); and, (iii) Southern and Western states and UTs (**Box 1**).

Box 1 **Regional classification of states.**

The three regions were:

- **Empowered Action Group (EAG) States** (Rajasthan, Madhya Pradesh, Chattisgarh, Odisha, Jharkhand, Bihar, Uttar Pradesh and Uttarakhand) **and North–Eastern (NE) States** (Sikkim, Assam, Meghalaya, Tripura, Mizoram, Manipur, Nagaland, Arunachal Pradesh); (The Government of India has identified eight states with poor health and development indicators as EAG states for focused action. EAG and NE states share similarities in MNCHN contexts and program performance);
- **Northern states and Union territories** (Jammu & Kashmir, Punjab, Himachal Pradesh, Haryana, Chandigarh, Delhi, and West Bengal); and
- **States and Union Territories in Southern and Western part of the country** (Kerala, Tamil Nadu, Karnataka, Andhra Pradesh and Telangana, Maharashtra, Gujarat, Goa, Puducherry).

## RESULTS

Of the 212 experts requested to contribute RIs, 90 (participation rate: 42.5%; 75 from the nation-wide network and 15 from the RSC) contributed 596 RIs. These were refined onto 648 RQs and finally consolidated as 101 ROs. ROs spanned across the four domains of research (d^4^ domains: description, discovery, development, and delivery). The description domain included 32 ROs (31.7%), 27 ROs (26.7%) were within development and another 27 ROs (26.7%) within delivery and only 2 (2%) within the discovery domain. The remaining 16 (15.8%) were classified within multiple domains, the majority of which including delivery (n = 13) or development (n = 8).

The 101 ROs were scored by 233 experts; 69 belonged to the EAG & NE region, 68 to the Northern region and 96 to the Southern and Western region. The gender and age distribution of the scorers, nationally and by region, is provided in **Table 1**.

**Table 1:** Gender and age distribution of scorers nationally and regionally

Characteristic		National (N = 233)						Empowered Action Group and North Eastern States (N = 69)						Northern States and Union Territories (N = 68)						Southern and Western States and Union Territories (N = 96)					
**ANS (N = 99)**	**EQU (n = 89)**	**REL (n = 88)**	**INN (n = 103)**	**INV (n = 87)**	**TOTAL (N = 233)**	**ANS (n = 29)**	**EQU (n = 25)**	**REL (n = 27)**	**INN (n = 27)**	**INV (n = 30)**	**TOTAL (N = 69)**	**ANS (n = 30)**	**EQU (n = 30)**	**REL (n = 24)**	**INN (n = 26)**	**INV (n = 26)**	**TOTAL (N = 68)**	**ANS (n = 40)**	**EQU (n = 34)**	**REL (n = 37)**	**INN (n = 50)**	**INV (n = 31)**	**TOTAL (N = 96)**
**Gender**	FEMALE	22	22	23	20	27	57	5	7	6	2	8	14	6	8	6	3	9	16	11	7	11	15	10	27
	MALE	77	67	65	83	60	176	24	18	21	25	22	55	24	22	18	23	17	52	29	27	26	35	21	69
**Age** **(in years)**	Mean ± SD	48.42 ± 11.05						50.19 ± 10.52						49.49 ± 10.13						46.39 ± 11.81					
	Median (IQR)	50(18)						52(17)						51(17)						45.5(22)					
	Min-Max	28-71						29-71						31-71						28-71					

ANS – answerability, EQU – equity, REL – relevance, INN – innovation, INV – innovation and out-of-box thinking and investment in research, SD – standard deviation, IQR – inter-quartile range, Min-Max – minimum-maximum

Table S1 in **Online Supplementary Document** shows the complete list of ranks, wRPS and AEA scores at the national and regional level for all 101 research priorities. Nationally, wRPS ranged from 0.923 to 0.512 and AEA ranged from 0.921 to 0.563.

The top 10 research priorities nationally, by domain, unweighted criterion scores, their wRPS and AEA are displayed in **Table 2**. All of the top 10 research priorities fell into either delivery (n = 5), development (n = 2) or a combination of delivery and development (n = 3). Nationally, the top research priorities were in the areas of cost-effectiveness, feasibility, barriers, and challenges in delivering interventions or programmes (numbers 1, 7, 9), monitoring and evaluation or surveillance (numbers 2, 8, 10), managing conditions through developing new technologies (numbers 3, 5, 6), and behaviour change (number 4).

**Table 2:** Top 10 Research Priorities for Child Health in India, Nationally, by domain and with ranks, unweighted scores* in each criterion, overall weighted research priority scores (wRPS)*, and Average Expert Agreement (AEA)

National Rank	Research priority	Domain(s)	Relevance	Answerability	Equity	Innovation	Investment in Research	National wRPS	AEA
1.	Develop locally relevant cost-effective strategies to expand the coverage of universal immunisation program (UIP) by reaching segments of populations that are traditionally left out (address system^1^ and community^2^ challenges)	Dev	0.94	0.95	0.99	0.84	0.88	0.92	0.92
	^1^Vaccine preventable diseases (VPD) epidemiology, system capacity, cold chain, safety surveillance								
	^2^Hesitancy, drop-out, outreach strategies, knowledge, attitudes, and practices (KAP) of care provider, community and clients								
2.	Improving administrative data quality and strengthening data-driven child health program monitoring, action, and accountability and primary health care (PHC) and district levels (eg, line listing of households with children with neuro-developmental disability (NDD), use of information and communication technologies (ICT), develop novel indicators).	Del	0.90	1.00	0.94	0.84	0.94	0.92	0.92
3.	Development and validation of low-cost technologies for screening, referral, and management of childhood pneumonia and acute respiratory infections (ARI) in the community and at various levels of health care (eg, mHealth, point-of-care diagnostics & therapeutics, management protocols, etc.).	Dev. & Del.	0.92	0.98	0.93	0.88	0.86	0.92	0.92
4.	Strategies to promote water, sanitation, and hygiene practices in the community to improve child health and nutrition.	Del.	0.93	1.00	0.89	0.88	0.85	0.92	0.91
5.	Development of cost-effective, feasible, validated point-of-care diagnostics for malaria in children for use at community and different levels of health care.	Dev. & Del	0.97	0.95	0.93	0.84	0.85	0.92	0.91
6.	Development of evidence-based guidelines for rational use of antibiotics for childhood morbidities in India: choice of antibiotics; route and delivery systems (eg, nebulizers); duration of therapy; monitoring criteria; adjunct therapies.	Dev.	0.96	0.99	0.83	0.87	0.91	0.91	0.91
7.	Development of an integrated child health program for improving quality of life of children: challenges and barriers; strategies to overcome; feasibility across the country; effectiveness; cost-effectiveness.	Dev. & Del.	0.93	0.96	0.92	0.91	0.78	0.91	0.90
8.	Establishing an effective and sustainable VPD surveillance program (especially measles and rubella, pneumonia and diarrhoea) in India [eg, defining syndromes (fever and rash) and program thresholds, forging public private partnerships (PPPs), building upon polio infrastructure, using technology such as mHealth, GIS, etc.).	Del.	0.92	0.97	0.91	0.87	0.84	0.91	0.90
9.	Identifying cost-effective strategies for supplementation of micronutrients and probiotics to prevent and control childhood diarrhoea, pneumonia and other infections.	Del.	0.91	0.98	0.91	0.85	0.85	0.90	0.90
10.	To establish a nation-wide multicentric antimicrobial surveillance and antibiotic stewardship program for infectious morbidities during childhood.	Del.	0.97	0.94	0.82	0.89	0.85	0.90	0.89

ANS – answerability, EQU – equity, REL – relevance, INN – innovation, INV – innovation and out-of-box thinking and investment in research, SD – standard deviation, IQR – inter-quartile range, Min-Max – minimum-maximum, Del – delivery, Dev – development, Disc – discovery, Desc – description

*Unweighted scores presented to explore researchers’ prioritisation and optimism of criteria relative to research priorities prior to weights being applied.

†Weighted RPS (wRPS) calculated by applying Larger Reference Group (LRG) weights (relevance = 0.254; answerability = 0.192; equity = 0.194; innovation and out of box thinking = 0.199; investment in research = 0.161) to unweighted scores and adding.

**Table 3****,** **Table 4** and **Table 5** display the top 10 research priorities for different regions of the country. Each table provides the RO list in descending order as per its priority score. The tables also provide the d^4^ domains, unweighted scores in each criterion, wRPS and AEA. The consistent feature was the emergence of implementation issues as the most important research options in the form of development and delivery with some variations across the regions. In North India and UTs, the top research priorities focused on better management of sickness, implementation of new point of care diagnostic tools (numbers 1, 2, 3, 4), monitoring and evaluation or surveillance (numbers 5, 9), program delivery and cost-effectiveness (numbers 7, 8, 10), and communications (number 6).

**Table 3:** Top 10 Research Priorities for Child Health in the Empowered Action Group and North-Eastern states, by domain and with regional ranks, unweighted scores* in each criterion, weighted research priority scores (wRPS), and average expert agreement (AEA)

Regional rank	Research priority	Domain(s)	Relevance	Answerability	Equity	Innovation	Investment in research	Regional wRPS	AEA
1.	Undertake systematic child health policy analysis for identifying strengths and gaps and developing policy guidance to meet the Sustainable Development Goals (SDGs).	Desc & Del	1.00	1.00	1.00	0.93	0.95	0.98	0.98
2.	Improving administrative data quality and strengthening data-driven child health program monitoring, action, and accountability and primary health centres (PHC) and district levels (eg, line listing of households with children with neuro-developmental delay (NDD), use of information communication technologies (ICT), develop novel indicators).	Del	0.94	1.00	0.95	0.95	1.00	0.96	0.97
3.	Development of evidence-based guidelines for rational use of antibiotics for childhood morbidities in India: choice of antibiotics; route and delivery systems (eg, nebulizers); duration of therapy; monitoring criteria; adjunct therapies.	Dev	1.00	1.00	0.81	1.00	0.96	0.96	0.95
4.	Development of cost-effective, feasible, validated point-of-care diagnostics for malaria in children for use at community and different levels of health care.	Dev & Del	1.00	0.96	0.95	0.96	0.85	0.95	0.94
5.	Development of portable water purifiers and recyclers for generating potable water and household levels.	Dev	0.94	1.00	0.95	0.94	0.91	0.95	0.95
6.	Integrate, revise, and evaluate curriculums for comprehensive skill building and their retention for health personnel involved in child health and nutrition services and all levels of care.	Del	0.95	1.00	0.86	0.95	0.95	0.94	0.94
7.	Development and validation of low-cost technologies for screening, referral, and management of childhood pneumonia and ARI in the community and at various levels of health care (eg, mHealth, point-of-care diagnostics & therapeutics, management protocols, etc.).	Dev	0.91	1.00	1.00	0.92	0.88	0.94	0.94
8.	Impact, process, and economic evaluation of the National Vector Borne Disease Control Program in the context of improving child health.	Del	0.95	0.96	0.90	0.90	1.00	0.94	0.94
9.	Identifying barriers and strategies to overcome and achieve Indian Public Health Standards (IPHS) benchmarks at primary and secondary level health facilities.	Desc& Del	0.88	1.00	0.94	1.00	0.90	0.94	0.94
10.	Strategies to engage the community and its resources (organizations, personnel) in improving the quality and outcome of the community-based management of childhood morbidities.	Dev	0.89	1.00	0.91	0.96	0.95	0.94	0.94

ANS – answerability, EQU – equity, REL – relevance, INN – innovation, INV – innovation and out-of-box thinking and investment in research, SD – standard deviation, IQR – inter-quartile range, Min-Max – minimum-maximum, Del – delivery, Dev – development, Disc – discovery, Desc – description

*Unweighted scores presented to explore researchers’ prioritisation and optimism of criteria relative to research priorities prior to weights being applied.

†Weighted RPS (wRPS) calculated by applying Larger Reference Group (LRG) weights (relevance = 0.254; answerability = 0.192; equity = 0.194; innovation and out of box thinking = 0.199; investment in research = 0.161) to unweighted scores and adding.

**Table 4:** Top 10 Research Priorities for Child Health in the Northern states and Union Territories, by domain and with regional ranks and unweighted scores* in each criterion, weighted research priority scores (wRPS), and average expert agreement (AEA)

Regional rank	Research priority	Domain(s)	Relevance	Answerability	Equity	Innovation	Investment in research	Regional wRPS	AEA
1.	Development and validation of low-cost technologies for screening, referral, and management of childhood pneumonia and acute respiratory infection (ARI) in the community and at various levels of health care (eg, mHealth, point-of-care diagnostics & therapeutics, management protocols, etc.).	Dev	0.95	0.96	0.92	0.95	0.91	0.94	0.94
2.	Identifying interventions to prevent/minimize development of adverse cardiometabolic and neurodevelopmental outcomes in low birthweight (LBW) [preterm and small for gestational age (SGA)] babies.	Dev	1.00	1.00	0.89	0.88	0.84	0.93	0.92
3.	Development of cost-effective, feasible, validated point-of-care diagnostics for malaria in children for use at community and different levels of health care.	Dev & Del	1.00	0.92	0.96	0.74	0.91	0.91	0.91
4.	Developing novel, cost-effective therapeutic regimens for treatment of resistant childhood malaria.	Dev	0.89	0.96	0.90	0.90	0.89	0.91	0.91
5.	Impact of Artemisinin Combination Therapy on malaria disease epidemiology and resistance patterns in India.	Del	0.95	1.00	1.00	0.68	0.89	0.91	0.90
6.	Identifying effective communication strategies (messages and channels) to improve awareness on child care and feeding practices during illness.	Dev & Del	0.95	0.92	0.88	0.91	0.83	0.90	0.90
7.	Development of an integrated child health program for improving quality of life of children: challenges and barriers; strategies to overcome; feasibility across the country; effectiveness; cost-effectiveness.	Dev & Del	0.90	1.00	0.92	0.86	0.83	0.90	0.90
8.	Develop locally relevant cost-effective strategies to expand the coverage of universal immunisation program (UIP) by reaching segments of populations that are traditionally left out (address system^1^ and community^2^ challenges):	Dev	0.90	0.96	0.96	0.78	0.91	0.90	0.90
	^1^Vaccine preventable diseases (VPD) epidemiology, system capacity, cold chain, safety surveillance								
	^2^hesitancy, drop-out, outreach strategies, knowledge, attitudes, and practices (KAP) of care provider, community and clients								
9.	Establishing an effective and sustainable VPD surveillance program (especially measles and rubella, pneumonia and diarrhoea) in India (eg, defining syndromes (fever and rash) and program thresholds, forging public private partnerships (PPPs), building upon polio infrastructure, using technology such as mHealth, GIS, etc.).	Del	0.90	0.96	0.88	0.91	0.83	0.90	0.90
10.	Improving diarrhoea control strategies in the public health system (eg,, oral rehydration solutions (ORS), zinc, water and sanitation hygiene (WASH), rational antibiotic and drug use).	Del	0.91	1.00	0.96	0.78	0.83	0.90	0.90

ANS – answerability, EQU – equity, REL – relevance, INN – innovation, INV – innovation and out-of-box thinking and investment in research, SD – standard deviation, IQR – inter-quartile range, Min-Max – minimum-maximum, Del – delivery, Dev – development, Disc – discovery, Desc – description

*Unweighted scores presented to explore researchers’ prioritisation and optimism of criteria relative to research priorities prior to weights being applied.

†Weighted RPS (wRPS) calculated by applying Larger Reference Group (LRG) weights (relevance = 0.254; answerability = 0.192; equity = 0.194; innovation and out of box thinking = 0.199; investment in research = 0.161) to unweighted scores and adding.

**Table 5:** *T*op 10 Research Priorities for Child Health in the Southern and Western states and Union territories, by domain and with regional ranks, unweighted scores^±^ in each criterion, weighted research priority scores (wRPS), and average expert agreement (AEA)

Regional rank	Research priority	Domain(s)	Relevance	Answerability	Equity	Innovation	Investment in research	Regional wRPS	AEA
1.	Develop locally relevant cost-effective strategies to expand the coverage of universal immunisation program (UIP) by reaching segments of populations that are traditionally left out (address system^1^ and community^2^ challenges):	Dev	1.00	0.92	1.00	0.85	0.86	0.93	0.93
	^1^Vaccine preventable diseases (VPD) epidemiology, system capacity, cold chain, safety surveillance								
	^2^Hesitancy, drop-out, outreach strategies, knowledge, attitudes, practices (KAP) of care provider, community and clients								
2.	Strategies to promote water, sanitation, and hygiene practices in the community to improve child health and nutrition.	Del	1.00	1.00	0.90	0.89	0.81	0.93	0.92
3.	Improving administrative data quality and strengthening data-driven child health program monitoring, action, and accountability and primary health centres (PHC) and district levels (eg, line listing of households with children with neuro-developmental delays (NDD), use of information communication technologies (ICT), develop novel indicators).	Del	0.93	1.00	0.92	0.84	0.95	0.93	0.93
4.	Impact evaluation of the universal immunisation program (UIP) with particular emphasis of recently introduced vaccines (eg, pentavalent measles, supplementary immunisation activities (SIAs), etc.)	Del	0.97	0.97	0.90	0.87	0.83	0.91	0.91
5.	Improving diarrhoea control strategies in the public health system (eg, oral rehydration solution (ORS), zinc, water and sanitation hygiene (WASH), rational antibiotic and drug use).	Del	0.94	0.97	0.87	0.83	0.93	0.91	0.91
6.	Development of an integrated child health program for improving quality of life of children: challenges and barriers; strategies to overcome; feasibility across the country; effectiveness; cost-effectiveness.	Dev & Del	1.00	0.89	0.96	0.89	0.75	0.91	0.90
7.	Identifying novel low-cost environmentally friendly strategies for control of vectors.	Dev	0.93	0.91	0.92	0.90	0.86	0.91	0.90
8.	Establishing an effective and sustainable VPD surveillance program (especially measles and rubella, pneumonia and diarrhoea) in India (eg, defining syndromes (fever and rash) and program thresholds, forging public private partnerships (PPPs), building upon polio infrastructure, using technology such as mHealth, GIS, etc.).	Del	0.94	0.94	0.90	0.87	0.85	0.90	0.90
9.	Strategies to engage the community and its resources (organizations, personnel) in improving the quality and outcome of the community-based management of childhood morbidities.	Dev	0.90	0.91	0.96	0.84	0.90	0.90	0.90
10.	Identifying cost-effective strategies for supplementation of micronutrients and probiotics to prevent and control childhood diarrhoea, pneumonia, and other infections.	Del	0.91	0.94	0.89	0.91	0.85	0.90	0.90

ANS – answerability, EQU – equity, REL – relevance, INN – innovation, INV – innovation and out-of-box thinking and investment in research, SD – standard deviation, IQR – inter-quartile range, Min-Max – minimum-maximum, Del – delivery, Dev – development, Disc – discovery, Desc – description

*Unweighted scores presented to explore researchers’ prioritisation and optimism of criteria relative to research priorities prior to weights being applied.

†Weighted RPS (wRPS) calculated by applying Larger Reference Group (LRG) weights (relevance = 0.254; answerability = 0.192; equity = 0.194; innovation and out of box thinking = 0.199; investment in research = 0.161) to unweighted scores and adding.

Among the top 10 research priorities for the Southern and Western States (**Table 5****)** development of low cost, environment friendly, and innovative interventions for vector control (number 7) and community engagement (number 9) were the novel and contextually important priority research areas.

The scorers from EAG and North-Eastern states **(****Table 3****)** favoured policy analysis specific to the SDGs, improving administrative data quality for decision making and better accountability and issues related to antimicrobial resistance as the top research priorities.

**Table 6** compares the rankings of the top 20 research priorities nationally with the regional rankings of those research priorities. There was divergence among priorities between the regions; 15 ROs each from North, and South & West regions appeared in the national list. EAG and NE states differed the most from the national list; the first priority of EAG and North-Eastern states did not appear in the national top 20 alongside 8 other such ROs. The Northern states and Union territories did not prioritise improving administrative data at the primary health centre (PHC) and district hospital level, examining barriers to achieving Indian Public Health Standards (IPHS) at primary and secondary level health facilities or community-based management of childhood morbidities generally, but rather prioritised research questions that were morbidity- or program-specific, with the exception of number 7. The Southern and Western states’ priorities were more aligned with the National priorities.

**Table 6:** Top 20 national research priorities with regional ranks

Priority research option	Rank			
**National**	**Empowered Action Group and North Eastern states**	**Northern States and Union Territories**	**Southern and Western States and Union Territories**
Develop locally relevant cost-effective strategies to expand the coverage of universal immunisation program (UIP) by reaching segments of populations that are traditionally left out (address system^1^ and community^2^ challenges):	1	11	8	1
^1^Vaccine preventable diseases (VPD) epidemiology, system capacity, cold chain, safety surveillance				
^2^hesitancy, drop-out, outreach strategies, knowledge, attitudes, practices (KAP) of care provider, community and clients				
Improving administrative data quality and strengthening data-driven child health program monitoring, action, and accountability and primary health centre (PHC) and district levels (eg, line listing of households with children with neuro-developmental delay (NDD), use of information communication technologies (ICT), develop novel indicators).	2	2	24	3
Development and validation of low-cost technologies for screening, referral, and management of childhood pneumonia and acute respiratory infection (ARI) in the community and at various levels of health care (eg, mHealth, point-of-care diagnostics & therapeutics, management protocols, etc.).	3	7	1	20
Strategies to promote water, sanitation, and hygiene practices in the community to improve child health and nutrition.	4	24	11	2
Development of cost-effective, feasible, validated point-of-care diagnostics for malaria in children for use at community and different levels of health care.	5	4	3	18
Development of evidence-based guidelines for rational use of antibiotics for childhood morbidities in India: choice of antibiotics; route and delivery systems (eg, nebulizers); duration of therapy; monitoring criteria; adjunct therapies.	6	3	19	11
Development of an integrated child health program for improving quality of life of children: challenges and barriers; strategies to overcome; feasibility across the country; effectiveness; cost-effectiveness.	7	31	7	6
Establishing an effective and sustainable VPD surveillance program (especially measles and rubella, pneumonia and diarrhea) in India (eg, defining syndromes (fever and rash) and program thresholds, forging public private partnerships (PPPs), building upon polio infrastructure, using technology such as mHealth, GIS, etc.).	8	22	9	8
Identifying cost-effective strategies for supplementation of micronutrients and probiotics to prevent and control childhood diarrhea, pneumonia and other infections.	9	30	12	10
To establish a nation-wide multicentric antimicrobial surveillance and antibiotic stewardship program for infectious morbidities during childhood.	10	23	14	12
Identifying novel low-cost environmentally friendly strategies for control of vectors.	11	25	18	7
Strategies to engage the community and its resources (organizations, personnel) in improving the quality and outcome of the community-based management of childhood morbidities.	12	10	37	9
Identifying barriers and strategies to overcome and achieve Indian Public Health Standards (IPHS) benchmarks at primary and secondary level health facilities.	13	9	42	14
Identifying effective communication strategies (messages and channels) to improve awareness on child-care and feeding practices during illness.	14	13	6	33
Impact, process, and economic evaluation of the National Vector Borne Disease Control Program in the context of improving child health.	15	8	33	28
Epidemiology, risk, and prognostic factors* of childhood pneumonia and ARI and their outcomes (including recurrence)	16	15	16	30
Impact evaluation of universal immunisation program (UIP) with particular emphasis of recently introduced vaccines (eg, pentavalent measles, supplementary immunisation activities (SIAs), etc.)	17	28	51	4
Impact of Artemisinin Combination Therapy on malaria disease epidemiology and resistance patterns in India.	18	20	5	44
Designing vaccination strategies for measles elimination in India in the context of epidemiological dynamics (age at immunization, number of doses, interval between doses, modes of vaccines delivery – routine vs SIA).	19	27	17	27
Improving diarrhea control strategies in the public health system (eg,, oral rehydration solution (ORS), Zinc, water and sanitation hygiene (WASH), rational antibiotic and drug use).	20	62	10	5

*Biological, genetic, maternal, familial, health, nutrition, socio-cultural, gender, demography, environment, economic, health system related.

Although the research priorities themselves are quite divergent, the type of research being prioritised was similar between regions. Many of the top research priorities across regions and nationally prioritised research on program delivery, including impact evaluation and cost-effectiveness studies on existing or new programs, improving monitoring and evaluation of existing conditions and programs and improving the management of morbidities.

## DISCUSSION

India is a vast and diverse country and so it might be inappropriate to consider single national-level research priorities, due to great levels of heterogeneity and inequity between states. Indeed, some authors have argued that reaching the MDGs was not possible due to this inequity [14]. Under-five mortality between states had an inverse relationship with economic transition status, and significant disparities existed in life expectancy [15]. The governance of health system also varied across the states [16]. Private health expenditure was high, and there have been disparities in States’ ability to finance health care. These were some of the factors that may have resulted in an imbalance on health spending between wealthier and poorer states, and management of the health system in general [15].

Because of this heterogeneity between health care access, governance, financing, and local socio-cultural practices, factoring context into this research priority setting exercise was of top importance in order to make this exercise as impactful as possible in achieving the SDGs. To account for the state-level heterogeneity, we aggregated research priorities for groups of states that were similar in economic transition level and governance of health systems. Similar to the recently published Million Deaths Study, Global Burden of Disease Study, and other analyses of disparities in service provision in India [2,4,5], the results of our research prioritisation exercise also demonstrated a need for regionally targeted programs.

The overall types of research being prioritised was similar across regions (ie, program delivery, monitoring and evaluation, and improved management of morbidities), but the content of the research options was different between regions. The EAG and North-Eastern states are among the least developed and was also the only region that prioritised a policy analysis reflecting the need for reviewing, refining and aligning programs and interventions to local requirements if they have to achieve SDGs in time. The other high priority RO from this region was the need for strengthening administrative data (number 2). Valid and high quality administrative data encourages timely decision making for rational and specific action in addition to bringing accountability across the health system [17,18]. The EAG-NE region and Northern states have high burden of malaria and hence prioritized ROs addressing point-of-care diagnostics, regimens for treating malaria, issues of resistance and its changing epidemiology. The Southern and Western states were relatively better governed and covered by health facilities and it appeared that they were now focussing on prevention; regional priorities related to vaccines introduction, safety monitoring and identifying strategies to reach the unreached segments of the populations [16].

Prioritising health research and aligning these with program interventions will be important to make progress to reach the SDGs. Indeed, the recent Global Burden of Disease Study for India found that not only there were differences between states at different levels of economic transition, states that were further along in economic transition had higher burden of non-communicable diseases in contrast to rest of the states with persistence of higher burden of communicable diseases. However, grouping states simply due to economic status might miss some nuances. For example, Odisha and Jharkhand had the highest DALYs due to diarrhoea but lowest due to LRTIs as compared to rest of India [4]. The results of this Global Burden of Disease study illustrated the importance of targeting health interventions and research priorities as much as possible.

In our exercise, researchers gave most ROs high scores against the ‘answerability’ and lower score to “innovation” criterion. The LRG on the other hand allocated greater weight to relevance and innovation. Thus, the LRG weights were impactful in discriminating between ROs and helped in deciding the final priority listing. The vast majority (>70%) of CHNRI exercises have not employed a LRG, which has the benefit of including wider stakeholder values in calculating research priorities [12,19]. Yoshida and colleagues posit that this is due to difficulty obtaining a representative sample of stakeholders [20]. Our LRG was composed of policy makers, representatives from funding agencies, senior researchers, and program managers from state and central government.

The EAG and North-Eastern states was the only region to prioritise conducting a policy analysis in-line with the SDGs in their top 10; this was their top priority. It was important to note that while this exercise was conducted thinking forward to the SDGs, the SDGs had not yet been released during the first round of the CHNRI (generating the ideas), and had been released in the middle of the second round (scoring the ideas). Therefore, while the exercise had the lens to look forward to the SDGs, it was not informed by the wider SDG agenda, which unlike the 8 MDGs, are much broader, and are comprised of 17 goals with 169 targets [21]. Many of the ideas prioritised in the current CHNRI exercise related to SDG 3.2, which asks to end preventable deaths children under 5 to at least 25 per 1000 live births [21]. This goal is similar to MDG 4, which focused on reducing child mortality and related interventions and monitoring, but misses the wider SDG agenda of child, economic, environmental, and societal well-being. SDG 3.2 is a crucial goal for India in obtaining the SDGs [22].

Moreover, one major cause of mortality was missing from the regional and national prioritised lists. The Million Deaths Study had demonstrated that India had shown accelerated declines in pneumonia and diarrhoea, though these were still major contributors to child deaths. However, deaths from injuries, including falls and drowning, have risen to the third most common cause of death in children, and is responsible for almost 70 000 deaths [2]. In an analysis including all ages, a separate paper showed that DALYs from injuries increased across all economic transition state groups in India [4]. Neither regions nor the overall country prioritised an intervention, program, or data collection mechanism for deaths due to injuries.

The d^4^ domains were independently assigned to every RO by three reviewers (KW, AM, and HG) and matched; disagreement was sorted by a senior researcher (NA) and through consensus with the reviewers. However, the domains overlapped and therefore, categorization of some of the research options was liable to subjective variation in interpretation of domains.

## CONCLUSIONS

This research priority exercise mobilised hundreds of researchers, funders, program managers from central and state governments, and policy makers and was part of a larger research priority setting exercise for maternal, child, newborn health, and maternal and child nutrition. Simply having research priorities are not enough; they must be implemented. The ICMR launched a grants programme for Implementation Research on Maternal and Child Health in spring 2017 to pursue the research priorities identified by this umbrella exercise. Ultimately, we hope that the outcome of this exercise and the joint ICMR grant programme shall positively impact child health in India, and accelerate India’s journey towards achieving the SDGs.

## Additional material

Online Supplementary Document
