# Supplementary material for: Setting priorities in child health research in India for 2016-2025: a CHNRI exercise undertaken by the Indian Council for Medical Research and INCLEN Trust
Source: J Glob Health. 2019 Oct 22;9(2):020701. doi: 10.7189/jogh.09.020701 (PMC6818639; doi:10.7189/jogh.09.020701)
Supplement: Online Supplementary Document [file jogh-09-020701-s001.pdf]

**Supplementary Table 1**. *Top 10 Research Priorities for Child Health in India, Nationally, by domain and with ranks, showing both unweighted and weighted scores± in each criterion, overall weighted research priority scores (wRPS)*, and Average Expert Agreement (AEA)*

| National Rank | Research Priority | Domain(s) | Relevance | Answerability | Equity | Innovation | Investment in Research | National wRPS | AEA |
|---|---|---|---|---|---|---|---|---|---|
| 1. | Develop locally relevant cost-effective strategies to expand the coverage of universal immunisation program (UIP) by reaching segments of populations that are traditionally left out (address system[1] and community[2] challenges)<br>[1]Vaccine Preventable Diseases (VPD) epidemiology, system capacity, cold chain, safety surveillance<br>[2]hesitancy, drop-out, outreach strategies, knowledge, attitudes, and practices (KAP) of care provider, community and clients | Dev | 0.94 (0.18) | 0.95 (0.19) | 0.99 (0.17) | 0.84 (0.14) | 0.88 (0.24) | 0.92 | 0.92 |
| 2. | Improving administrative data quality and strengthening data-driven child health program monitoring, action, and accountability and primary health care (PHC) and district levels (e.g. line listing of households with children with neuro-developmental disability (NDD), use of information and communication technologies (ICT), develop novel indicators). | Del | 0.90 (0.19) | 1.00 (0.18) | 0.94 (0.17) | 0.84 (0.15) | 0.94 (0.23) | 0.92 | 0.92 |
| 3. | Development and validation of low-cost technologies for screening, referral, and management of childhood pneumonia and acute respiratory infections (ARI) in the community and at various levels of health care (e.g. mHealth, point-of-care diagnostics & therapeutics, management protocols, etc.). | Dev. & Del. | 0.92 (0.19) | 0.98 (0.18) | 0.93 (0.18) | 0.88 (0.14) | 0.86 (0.23) | 0.92 | 0.92 |
| 4. | Strategies to promote water, sanitation, and hygiene practices in the community to improve child health and nutrition. | Del. | 0.93 (0.18) | 1.00 (0.18) | 0.89 (0.17) | 0.88 (0.14) | 0.85 (0.25) | 0.92 | 0.91 |
| 5. | Development of cost-effective, feasible, validated point-of-care diagnostics for malaria in children for use at community and different levels of health care. | Dev. & Del | 0.97 (0.19) | 0.95 (0.18) | 0.93 (0.17) | 0.84 (0.14) | 0.85 (0.24) | 0.92 | 0.91 |
| 6. | Development of evidence-based guidelines for rational use of antibiotics for childhood morbidities in India: choice of antibiotics; route and delivery systems (e.g. nebulizers); duration of therapy; monitoring criteria; adjunct therapies. | Dev. | 0.96 (0.19) | 0.99 (0.16) | 0.83 (0.17) | 0.87 (0.15) | 0.91 (0.24) | 0.91 | 0.91 |
| 7. | Development of an integrated child health program for improving quality of life of children: challenges and barriers; strategies to overcome; feasibility across the country; effectiveness; cost effectiveness. | Dev. & Del. | 0.93 (0.18) | 0.96 (0.18) | 0.92 (0.18) | 0.91 (0.13) | 0.78 (0.24) | 0.91 | 0.90 |

| National Rank | Research Priority | Domain(s) | Relevance | Answerability | Equity | Innovation | Investment in Research | National wRPS | AEA |
|---|---|---|---|---|---|---|---|---|---|
| 8. | Establishing an effective and sustainable Vaccine Preventable Disease surveillance program (especially measles and rubella, pneumonia and diarrhoea) in India [e.g. defining syndromes (fever and rash) and program thresholds, forging public private partnerships (PPPs), building upon polio infrastructure, using technology (mHealth, GIS, etc.)]. | Del. | 0.92 (0.19) | 0.97 (0.18) | 0.91 (0.17) | 0.87 (0.14) | 0.84 (0.23) | 0.91 | 0.90 |
| 9. | Identifying cost-effective strategies for supplementation of micronutrients and probiotics to prevent and control childhood diarrhoea, pneumonia and other infections. | Del. | 0.91 (0.19) | 0.98 (0.17) | 0.91 (0.17) | 0.85 (0.14) | 0.85 (0.23) | 0.90 | 0.90 |
| 10. | To establish a nation-wide multicentric antimicrobial surveillance and antibiotic stewardship program for infectious morbidities during childhood. | Del. | 0.97 (0.18) | 0.94 (0.16) | 0.82 (0.18) | 0.89 (0.13) | 0.85 (0.25) | 0.90 | 0.89 |

±Unweighted scores presented to explore researchers' prioritisation and optimism of criteria relative to research priorities prior to weights being applied. Weighted scores are added together to calculate RPS, thereby they appear much lower than unweighted scores

*Weighted RPS (wRPS) calculated by applying Larger Reference Group (LRG) weights (relevance = 0.254; answerability = 0.192; equity = 0.194; innovation and out of box thinking = 0.199; investment in research = 0.161) to unweighted scores and adding.

Domains: Delivery (Del); Development (Dev); Discovery (Disc); Description (Desc)
